# Voltage-Gated Calcium Channels and α-Synuclein: Implications in Parkinson’s Disease

**DOI:** 10.3389/fnmol.2019.00237

**Published:** 2019-10-09

**Authors:** Emmanouela Leandrou, Evangelia Emmanouilidou, Kostas Vekrellis

**Affiliations:** ^1^Center for Basic Research, Biomedical Research Foundation Academy of Athens, Athens, Greece; ^2^Laboratory of Biochemistry, Department of Chemistry, National and Kapodistrian University of Athens, Athens, Greece

**Keywords:** alpha-synuclein, Parkinson’s and related diseases, protein aggregation, secretion, calcium, voltage gated Ca^2+^ channel, neurodegeneration

## Abstract

Alpha-synuclein (α-syn) is biochemically and genetically linked to Parkinson’s disease (PD) and other synucleinopathies. It is now widely accepted that α-syn can be released in the extracellular space, even though the mechanism of its release is still unclear. In addition, pathology-related aggregated species of α-syn have been shown to propagate between neurons in synaptically connected areas of the brain thereby assisting the spreading of pathology in healthy neighboring neuronal cells. In neurons, calcium channels are key signaling elements that modulate the release of bioactive molecules (hormones, proteins, and neurotransmitters) through calcium sensing. Such calcium sensing activity is determined by the distinct biophysical and pharmacological properties and the ability of calcium channels to interact with other modulatory proteins. Although the function of extracellular α-syn is currently unknown, previous work suggested the presence of a calcium-dependent mechanism for α-syn secretion both *in vitro*, in neuronal cells in culture, and also *in vivo*, in the context of a *trans*-neuronal network in brain. Mechanisms regulating extracellular α-syn levels may be of particular importance as they could represent novel therapeutic targets. We discuss here how calcium channel activity may contribute to α-syn aggregation and secretion as a pathway to disease progression in synucleinopathies.

## Introduction

The affected neurons in Parkinson’s disease (PD) brains contain dense filamentous inclusions called Lewy bodies that primarily consist of the presynaptic protein α-synuclein (α-syn), a small neuronal protein that is abundant throughout the central nervous system under normal conditions (Goedert et al., 2017). The causal role of α-syn, a protein physiologically present in all neurons, in the pathogenesis of PD is further highlighted by human genetic studies that have linked multiplications and coding-region point mutations of the *SNCA* gene encoding for α-syn with familial PD (Houlden and Singleton, 2012). Increased levels of aggregated α-syn species have been strongly correlated with neuronal cell death partly due to a potent impairment of the degradation capacity of cellular proteolytic systems. Apart from acting in the cytoplasm, α-syn is normally secreted in the interstitial fluid (ISF) of the brain and can be taken up by neuronal cells (reviewed in Stefanis et al., 2019). High burden of extracellular α-syn has been shown to promote the production and transfer of pathology-linked, possibly hyper-aggregated, α-syn material. Under pathological conditions, such as PD, these pathologically relevant species of α-syn have the ability to propagate along interconnected neuronal networks leading to a progressive degeneration of neuronal function and ultimately cell death (Bieri et al., 2018).

It is now established that α-syn binds membranes with high affinity and this binding determines its functions especially at the synaptic sites. In particular, α-syn has been implicated in the process of neurotransmitter release where a number of potential roles of the protein in the maturation of synaptic vesicles, vesicle docking, priming and fusion have been proposed. The suggested mechanisms involve in part a direct or indirect interaction with the SNARE complex, but whether α-syn promotes or disrupts the formation of SNARE complex is still under debate (reviewed in Huang et al., 2019). How aggregated α-syn assemblies interfere with neurotransmitter release thereby affecting the functionality of neuronal synapses also remains elusive. In PD brains, deposits of small oligomers/aggregates of α-syn can be detected in the presynaptic sites prior to the formation of Lewy bodies and synaptic deficits seem to precede cell death in the course of the disease (Calo et al., 2016). The presence of abnormal α-syn assemblies extracellularly could modify neurotransmission and drastically affect the network dynamics of interconnected microcircuits and enhance the progression of disease pathology by facilitating the cell-to-cell transfer of pathological synuclein species.

Aggregation in the extracellular space may be favored by high local accumulation of α-syn. As such, the regulation of extracellular α-syn levels could be used therapeutically providing we understand the mechanism(s) of α-syn secretion and how this is triggered. Previous work indicated the presence of a Ca^2+^-dependent mechanism for α-syn secretion both *in vitro*, in neuronal cells in culture, and also *in vivo*, in the context of a *trans*-neuronal network in mouse brain, where α-syn release is mediated by presynaptic Ca^2+^ channels (Emmanouilidou et al., 2010, 2016). It is possible that abnormal function of the specific Ca^2+^ channel(s) regulating α-syn secretion could alter the levels of α-syn released to the extracellular space in a manner that favors the local aggregation of the protein at least in certain extra-synaptic sites. The aggregated assemblies may then be internalized by neighboring neurons acting as seeds to recruit the intracellular α-syn and facilitate the templating and subsequent release of pathological α-syn species by a cell-to-cell propagation mechanism.

The main presynaptic Ca^2+^ channels are the voltage-gated calcium channels (VGCCs) and the ligand-gated ion channels. The VGCCs consist of ten calcium channel isoforms, nine of which are widely expressed in the nervous system. VGCCs are responsible for calcium influx thereby controlling neuronal calcium homeostasis. As summarized in Table 1, dysregulation of VGCCs expression or activity has been linked with many neurological disorders such as Alzheimer’s disease, Parkinson’s disease, Multiple Sclerosis, iron, and zing neurotoxicity (Cataldi, 2013). In this review we focus on the role of VGCCs in PD and especially in neuronal degeneration that is apparent in PD. Up to date, there is no direct evidence that presynaptic calcium channels regulate α-syn propagation. In the paragraphs that follow, we try to analyze the role of Ca^2+^ signaling in α-syn aggregation and secretion, as they depict the major precursor mechanisms for α-syn propagation.

**TABLE 1:** Voltage-gated calcium channel blockers and their effect on PD pathophysiology.

**VGCCs**	**Blocker**	**Experimental model**	**Effect**	**References**
N-type P/Q-type	ω-conotoxin ω-agatoxin	Sprague–Dawley rats	Decreased the dopamine release from striatal terminals	Bergquist et al., 1998
R-type	SNX-482	Sprague–Dawley rats	Decreased the somatodendritic DA release in SN	Bergquist and Nissbrandt, 2003
T-type	Ni^2+^ mifebradil	STN slices + Wistar rats	Reduced the burst activity in STN neurons and improved the locomotor deficits in 6-OHDA lesioned rats	Tai et al., 2011
	ML218	iPSCs derived from dopaminergic neurons of PARK-2 patients	Ameliorated the effect of rotenone treatment by rescuing the neuronal apoptotic phenotype	Tabata et al., 2018
N-type	ω-conotoxin	Rat primary cortical neurons	Diminished the elevation of intracellular calcium and dopamine release that was triggered after extracellular α-synuclein application	Ronzitti et al., 2014
L-type N-type	Nifedipine ω-conotoxin	SH-SY5Y cells	Diminished the elevation of intracellular calcium that was observed when extracellular α-synuclein was applied to the cells	Melachroinou et al., 2013
	Isradipine	Adult brain slices + C57BL/6 mice	Reversed the rotenone and MPTP-induced TH-loss and motor deficits	Chan et al., 2007
	Nimodipine	Primary dopaminergic neurons	Reduced the increased levels of cytosolic dopamine that are observed after L-DOPA administration	Mosharov et al., 2009
	Isradipine	C57B1/6 mito-roGFP transgenic mice	Reduced the mitochondrial oxidant stress in SNc dopaminergic neurons	Guzman et al., 2010
	Isradipine	C57BL/6 mice	Increased the survival of SNc dopaminergic cells after 6-OHDA- induced degeneration	Ilijic et al., 2011
L-type	Isradipine Nimodipine	Primary dopaminergic neurons	Prevented the MPP^+^-induced intracellular calcium elevation in SN but not in VTA.	Lieberman et al., 2017
	Isradipine	6-OHDA-treated mice	Failed to achieve neuroprotection of SNc neurons, due to low selectivity for Cav1.3 VGCCs	Ortner et al., 2017
	Isradipine	TH-mito-roGFP transgenic mice	Decreased mitochondrial oxidant stress was achieved by reducing Ca^2+^ oscillations in SNc	Guzman et al., 2018
	Isradipine	Ventral mesencephalic primary neurons	Reversed the clustering of a-synuclein positive vesicles and α-synuclein aggregation that is observed after dopamine administration	Lautenschläger et al., 2018

## Calcium and α-Synuclein

Calcium homeostasis is important for the maintenance of neuronal integrity, since it is involved in synaptic transmission, neuronal plasticity, and cell survival. Activation of calcium signaling cascades is a result of intracellular calcium elevation, either via calcium influx or via calcium release from intracellular stores, such as the endoplasmic reticulum (ER) (reviewed in Yang et al., 2019). Among the different events that trigger α-syn pathology, the disruption of calcium homeostasis stands out as a possible mediator of aggregation and abnormal secretion of α-syn.

α-syn aggregation can be enhanced by alterations in intracellular calcium concentration in a direct or indirect manner. It has been shown that a transient increase of intracellular calcium leads to an increase in α-syn aggregates in human cell lines expressing α-syn (Nath et al., 2011; Follett et al., 2013). The mechanism through which calcium promotes α-syn aggregation is not clear. It has been shown that subsequent to calcium binding, the calcium-binding protein, calmodulin (CaM), changes its conformation and binds to α-syn inducing α-syn fibrillization (Lee et al., 2002; Martinez et al., 2003). CaM along with calbindin are calcium-binding proteins that are used by the cell as buffering proteins to maintain intracellular calcium homeostasis. The interplay between the presence of calbindin and the progression of PD was initially proposed after the discovery of a subgroup of calbindin-positive dopaminergic neurons that exhibited less pathologic features compared to calbindin-negative dopaminergic neurons in rat substantia nigra pars compacta (SNpc) and postmortem human brain material from PD patients (Gerfen et al., 1985; Yamada et al., 1990). Furthermore, a study in human brain tissues from dementia with Lewy bodies (DLB) patients showed that the Lewy bodies were exclusively formed in calbindin-negative neurons, a result that was confirmed using the mouse rotenone model, where α-syn aggregation was evident primarily in calbindin-negative neurons (Rcom-H’cheo-Gauthier et al., 2016). In the same line of evidence, striatal administration of a calbindin-expressing adenoviral vector in macaque monkeys protected the nigrostriatal dopamine system from 1-methyl-4-phenyl-1,2,3,6-tetrahydropyridine (MPTP)-induced degeneration (Inoue et al., 2019). These data suggest that α-syn aggregates are more prone to be formed in neurons with decreased calcium buffering capacity possibly due to irregular intracellular calcium levels.

Apart from calcium binding proteins, calcium-dependent proteases, such as calpain, seem to play a role in α-syn aggregation. In particular, increase in intracellular calcium concentrations lead to pathologically increased calpain activity. Mice deficient for calpastatin, a calpain-specific inhibitor, that were crossed with A30P α-syn expressing mice, exhibited higher calpain activity and increased α-syn aggregated species (Diepenbroek et al., 2014).

Finally, it has been proposed that calcium binds directly to the C-terminal of α-syn (Nielsen et al., 2001). Using time-resolved circular dichroism spectroscopy and infrared spectroscopy, it has been shown that calcium binding leads to exposure of the non-amyloid component (NAC) domain of the monomeric protein promoting the formation of β-sheet structures and thus accelerating the formation of α-syn aggregates (Han et al., 2018). In a different point of view, application of recombinant α-syn monomers or oligomers to primary neuronal cultures induced an increase in cytosolic transient [Ca^2+^] linking α-syn-induced neurotoxicity with increased intracellular calcium signaling (Angelova et al., 2016). By using the planar lipid bilayer approach, the authors concluded that the interaction of α-syn with the plasma membrane could facilitate calcium influx via affecting membrane permeability (Angelova et al., 2016). Alternatively, it has been proposed that α-syn aggregates can bind to the Ca^2+^ pump sarco/endoplasmic reticulum Ca^2+^-ATPase (SERCA) on the endoplasmic reticulum modulating the intracellular calcium concentration. In this study, blockage of SERCA ameliorated the α-syn aggregated-induced cell death in neuronal cells in culture (Betzer et al., 2018).

Alpha-synuclein is considered to be secreted from neuronal cells though a stimulus-dependent mechanism that is regulated by the levels of intracellular Ca^2+^. It has been shown that an increase in intracellular calcium stimulate the secretion of α-syn in α-syn expressing SH-SY5Y cells (Emmanouilidou et al., 2010). Importantly, the secretion of α-syn in mouse striatum is thought to be regulated by the operation of presynaptic calcium channels as has been shown using a reverse microdialysis approach (Emmanouilidou et al., 2016). This is in accordance with a recent study that proposes, using a similar experimental setting, that the release of α-syn is dependent on neuronal activity *in vivo* (Yamada and Iwatsubo, 2018). The above studies support the idea that α-syn can be found in extracellular milieu not only as a monomer but also as an oligomeric conformer that may be internalized by neuronal cells.

**FIGURE 1:**
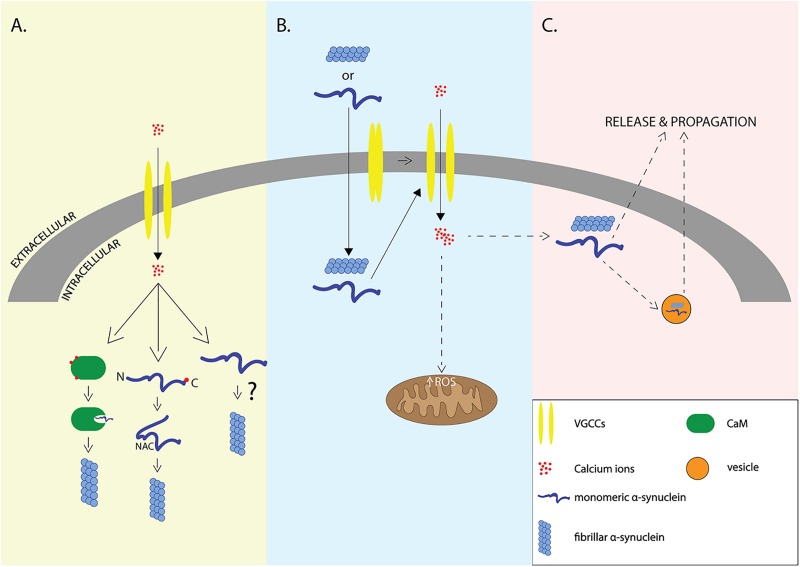
Interplay of α-syn and Ca^2+^. Elevation of intracellular Ca^2+^ levels through voltage-gated calcium channels (VGCCs) may lead to aggregation of α-syn via indirect interactions with calcium binding proteins such as Calmodulin, or via direct binding of Ca^2+^ to α-syn that leads to exposure of NAC domain **(A)**, elevation of intracellular amounts of α-syn may lead to VGCCs opening and calcium influx that results in increased formation of reactive oxygen species (ROS) and neurodegeneration **(B)**. It still remains unclear whether elevation of intracellular Ca^2+^ is the cause or the result of α-syn aggregation which promotes the release and propagation of α-syn -the release is accomplished either through exosomes or through other proposed secretory mechanisms **(C)**.

The work described so far supports a multifactorial relationship of α-syn with calcium signaling where elevations in intracellular Ca^2+^ can result in the aggregation and release of α-syn or vice versa (Figure 1).

## Channelopathies and Neurodegeneration in Pd

In search of the mechanisms of disease progression in PD, previous work has focused on the connection between α-syn secretion, neurodegeneration, and alterations in calcium influx, mainly via the VGCCs. In neurons, VGCCs are key signaling elements that modulate the release of bioactive molecules (hormones, proteins, and neurotransmitters) through Ca^2+^ sensing. Following neuronal spiking and K^+^ channels opening, VGCCs modulate Ca^2+^ influx during repolarization back to the resting membrane potential. In most cases, VGCCs open at high voltages and close slowly during repolarization, leaving a time window where calcium influx occurs. The amount of calcium ions that enter the cell depend on the duration of the spike. Neurons regulate the amount of calcium influx by reducing the duration of the spikes (less than 1 ms) and by expressing calcium binding proteins (reviewed in James Surmeier et al., 2012). The VGCCs are divided to high-voltage activated (HVA) channels, which are activated at high membrane depolarization, or low-voltage activated (LVA) channels, which open at voltages near the resting membrane potential (Armstrong and Matteson, 1985; Bean, 1985). The HVA channels include L-type(Cav1 family), P-, Q-, and R-type (Cav2 family) VGCCs, whereas LVA channels include only T-type channels (Cav3 family) (reviewed in Zamponi, 2016).

It is noteworthy that in PD degeneration occurs preferentially in SNc dopaminergic neurons, while ventral tegmental area (VTA)-residing dopaminergic neurons remain mostly unaffected. It is proposed that differences in the regulation of calcium balance in these two neuronal populations could contribute to the onset and progression of neurodegeneration in PD. In particular, the adult SNc neurons mostly rely on L-type VGCCs for their basal activity and, more specifically, on the Cav1.3 subtype of L-type channels that open at relatively negative membrane potential (Mercuri et al., 1994). SNc dopaminergic neurons also express calcium binding proteins at lower level compared to VTA dopaminergic neurons (Foehring et al., 2009). These differences seem to be developmentally coordinated; it has been shown that during the embryonic stage and until postnatal week 3, the SNc dopaminergic neurons rely mostly on voltage-dependent Na^+^ channels, until a developmental switch renders Cav1.3-VGCCs the primary voltage-gated channels responsible for their pacemaking activities (Chan et al., 2007). In support for a critical role of VGCCs in the susceptibility of dopaminergic neurons, application of nimodipine, a Cav1.2/Cav1.3 blocker, in cultured L-DOPA-treated midbrain neurons decreased the levels of cytosolic dopamine suggesting a role of L-type channels in dopamine metabolism and neuronal survival (Mosharov et al., 2009). In addition, the L-type VGCCs were shown to be responsible for the elevation of intracellular calcium in primary dopaminergic neurons exposed to MPP^+^. Such calcium elevation was not observed in α-syn knock-out cultures after MPP^+^ exposure, suggesting that α-syn is implicated in intracellular calcium changes under stress conditions (Lieberman et al., 2017). Interestingly, increased intracellular calcium in SNc neurons overexpressing α-syn led to increased mitochondrial oxidation and neurotoxicity in these neurons, but not VTA neurons, probably due to the fact that VTA neurons do not depend on Cav1.3 VGCCs for neuronal firing (Lieberman et al., 2017). In support to these observations, a recent study depicted the importance of a fine balance between intracellular α-syn and intracellular calcium. In mesencephalic neurons, calcium binding in α-syn mediates the localization of the protein in synaptic vesicles and, under conditions of increased calcium or α-syn, this localization promotes synaptic vesicle clustering and α-syn aggregation. In this system, isradipine treatment reversed α-syn aggregation and improved neuronal survival (Lautenschläger et al., 2018). Several other studies using pharmacological inhibition emphasized on the importance of L-type channels in degeneration. Blockage of L-type calcium channels with the L-type inhibitor isradipine significantly reduced mitochondrial oxidation, indicating that calcium influx via the L-type VGCCs during pacemaking plays an important role in mitochondrial oxidant stress (Guzman et al., 2010). Furthermore, systemic administration of isradipine protected the striatal dopaminergic terminals, as well as the somata of dopaminergic neurons, after intrastriatal injection of 6-hydroxydopamine (6-OHDA) in mice (Ilijic et al., 2011).

Apart from the degeneration of dopaminergic neurons in SNpc of PD patients, the pathology is also evident in nuclei of the brainstem and olfactory bulbs (Jellinger, 2008). Cholinergic neurons in the dorsal motor nucleus of the vagus (DMV) in the caudal medulla have been shown to exhibit early Lewy body formation (Braak and Del Tredici, 2009). Interestingly, these neurons are autonomous slow pacemakers receiving an increased intracellular calcium load via the L-type VGCCs and they express low levels of calcium binding proteins. To induce mitochondrial stress, Goldberg et al. (2012) diminished DJ-1 from cholinergic DMV neurons and showed that pharmacological blockage of L-type VGCCs ameliorates the induced mitochondrial stress.

Further addressing a key role of L-type channels in PD, examination of post mortem material has revealed significant differences in the expression levels of these channels in PD patients compared with healthy subjects suggesting a possible role of the L-type family in the process of neurodegeneration, probably, via increased calcium influx that may lead to excitotoxicity. Immunostaining for Cav1 channels revealed that the expression ratio of Cav1.3/Cav1.2 of Cav1 was increased in early-stage PD brains compared to healthy controls (Hurley et al., 2013). Furthermore, two independent drug epidemiological studies targeting Cav1.3 channels concluded that administration of dihydropyridines lead to a reduced risk of developing PD (Ritz et al., 2010; Pasternak et al., 2012). However, since both Cav1.2 and Cav1.3 channels have very similar structural and pharmacological properties, the selectivity of 1,4-dihydropyridines is very low and the application of these L-type inhibitors could also block Cav1.2 channels (Xu and Lipscombe, 2001). In this context, isradipine-a well-known anti-hypertensive drug- is currently in phase III clinical trials to determine whether it can be effective against the progression of PD (Biglan et al., 2017). Isradipine has previously been used against neurodegeneration, but its selectivity is still under debate (Ortner et al., 2017; Guzman et al., 2018). In general, the variability in the blocking properties of dihydropyridines during different membrane depolarization states of dopaminergic neurons raises concerns about the usage of these drugs for PD treatment, since higher doses of dihydropyridines might not be tolerated during long-term treatment (Ortner et al., 2017).

Specific VGCCs have been implicated in α-syn-induced neurotoxicity. In SH-SY5Y cells, treatment with either nifedipine or ω-conotoxin, specific inhibitors for the L-type and N-type VGCCs, respectively, diminished the intracellular calcium raise induced by extracellular α-syn (Melachroinou et al., 2013). In rat cortical neurons, Ronzitti et al. (2014) showed that the calcium influx following application of extracellular α-syn was abolished by ω-conotoxin, but not by nifedipine or ω-agatoxin (specific inhibitors for L-type and P/Q type, respectively) indicating a possible role for the N-type VGCCs on α-syn-induced calcium increase.

Finally, targeting of T-type VGCCs has recently been considered a neuroprotective strategy for neurodegeneration and, more specifically, PD (Kopecky et al., 2014; Yang et al., 2014). Tabata et al. (2018) highlighted the importance of T-type VGCCs using induced pluripotent stem cell (iPSC) dopamine neurons derived from PARK2 patients by applying pharmacological or genetic silencing of T-type channels. In this study, the specific T-type antagonist ML218 ameliorated the effects of rotenone-induced mitochondrial stress by rescuing the apoptotic phenotype thereby leading to neuroprotection. Similar results were obtained after silencing of T-type VGCCs with specific siRNAs, while overexpression of T-type VGCCs led to the opposite effects. It has also been suggested that T-type VGCCs play a role in locomotor deficits accompanied after 6-OHDA lesion in rats. Specifically, *in vitro* as well as *in vivo* studies on subthalamic nuclei (STN) neurons revealed that blockage of T-type channels by the T-type specific inhibitors, Ni^2+^ and mifebradil, reduced the pathologically increased oscillations of STN that may be responsible for the tremor and other motor deficits present in PD. The above finding was also confirmed by behavioral experiments, where 6-OHDA lesioned rats showed a significant improvement in open field locomotor test after direct microinjection of either Ni^2+^ of mifebradil in STN (Tai et al., 2011).

## Concluding Remarks

We can conclude that all different types of VGCCs have been implicated in the progressive neurodegeneration present in PD. This is highlighted by a plethora of studies in which specific VGCCs are pharmacologically targeted in dopaminergic neurons to assess their role in preserving normal dopamine release and promoting cell survival under conditions of cellular stress. Several parameters could contribute to the discrepancies observed among the different studies. The different model systems used, ranging from *in vitro* cell models, such as cell lines and primary neurons, to the living rodent brain, could affect the magnitude and interpretation of the effects observed following the pharmacological manipulation of each VGCC. It is also possible that the different α-syn species (monomers, oligomers, and fibrils) have the ability to act through independent molecular mechanisms to trigger alterations in intracellular calcium. Finally, the pharmacological inhibition of certain VGCCs could stimulate compensatory mechanisms in which other calcium channels operate synergistically to regulate calcium levels adding further complexity to the interpretation of the results obtained so far.

Calcium influx can trigger α-syn aggregation thus providing an alternative pathway to PD neurodegeneration. There is also evidence that VGCCs can facilitate α-syn secretion under normal or pathological conditions, even though the mechanism for the stimulation of this process is still elusive. Abnormal function of these specific VGCCs may cause local accumulation of aggregated α-syn material into the extracellular space which could be taken up by recipient neurons thereby promoting the cell-to-cell spreading of disease pathology. As such, VGCCs that regulate α-syn properties could indicate specific molecular pathways to target as alternative therapeutic approaches for PD.

## Author Contributions

All authors listed have made a substantial, direct and intellectual contribution to the work, and approved it for publication.

## Conflict of Interest

The authors declare that the research was conducted in the absence of any commercial or financial relationships that could be construed as a potential conflict of interest.

## Abbreviations

α-syn: alpha-synuclein
6-OHDA: 6-hydroxydopamine
CaM: calmodulin
DLB: dementia with Lewy bodies
DMV: dorsal motor nucleus of the vagus
ER: endoplasmic reticulum
HVA: high-voltage activated
iPSC: induced pluripotent stem cell
ISF: interstitial fluid
LVA: low-voltage activated
MPTP: 1-methyl-4-phenyl-1,2,3,6-tetrahydropyridine
NAC domain: non-amyloid component domain
PD: Parkinson’s disease
SERCA: sarco/endoplasmic reticulum Ca^2+^-ATPase
SNpc: substantia nigra pars compacta
STN: subthalamic nuclei
VGCCs: voltage-gated calcium channels
VTA: ventral tegmental area.
